# Growth patterns of the nasolabial region following unilateral cleft lip primary repair

**DOI:** 10.3389/fped.2023.1136467

**Published:** 2023-03-13

**Authors:** Yulang Xu, Ni Zeng, Jingtao Li, Qian Zheng, Bing Shi

**Affiliations:** State Key Laboratory of Oral Diseases, National Clinical Research Center for Oral Disease, and Department of Oral and Maxillofacial Surgery, West China School of Stomatology, Sichuan University, Chengdu, China

**Keywords:** unilateral cleft lip, primary repair, primary rhinoplasty, nasolabial region, growth patterns

## Abstract

Surgical correction is the optimal way of repairing a congenital cleft lip. Patients with this condition often undergo initial surgical treatment at an early age and achieve an acceptable outcome. However, their levels of satisfaction will decrease in later stages of life as facial growth and development will inevitably cause changes in long-term outcomes, especially in the nasolabial region. Therefore, it is important for surgeons to understand nasolabial development after primary treatment and tailor their surgical techniques appropriately. This review focuses on the growth patterns of the nasolabial region after primary repair, so as to provide references for operative strategy.

## 1. Introduction

Cleft lips (CLs) are the most common congenital deformities affecting the orofacial region; patients with CLs usually have significant orofacial deformities (1–3). Moreover, patients with a unilateral cleft lip and palate (UCLP) were detected with a more pronounced asymmetry than those with a bilateral cleft lip and palate (BCLP) (4). Significant differences between the cleft and the non-cleft sides existed only around the cleft but not in the broader regions of the maxillary complex (5). Therefore, the main orofacial deformities are manifested in soft tissue covering the nasolabial region (6), for example, a deviation of the columella toward the non-cleft side, widening of the nasal sill, displacement of the alar base, and flattening of the lower lateral cartilage (LLC) (7).

Deformities in soft tissue can be easily corrected compared with those in hard tissue (8). In order to improve the soft tissue profile, some researchers proposed that there was a close relationship among muscles in the nasolabial region, histologically and biomechanically, and the deformities of the cleft lip were the result of the joint actions of these muscles, which were caused by the uneven distribution of the nasolabial muscles (9–11).

Due to facial development and growth, the nasolabial morphology changes, eventually leading to significant residual asymmetry. Therefore, the main challenge in cleft lip primary reconstruction is to restore normal nasolabial morphology by taking into account various perspectives, with consideration given to the anticipated changes that occur over time (12). This places a great deal of responsibility on surgeons to accurately assess the anatomic deformity along with anticipated fourth-dimensional changes and improve and adjust their surgical strategy so as to optimize long-term postoperative outcomes (13, 14).

This review summarizes the growth patterns of the nasolabial region following unilateral cleft lip primary repair, in order to provide a reference for the surgical refinement of primary repair of CL so as to minimize facial asymmetry and guide secondary corrective surgery.

## 2. Landmarks and measurements of the nasolabial region

**Figure 1:**
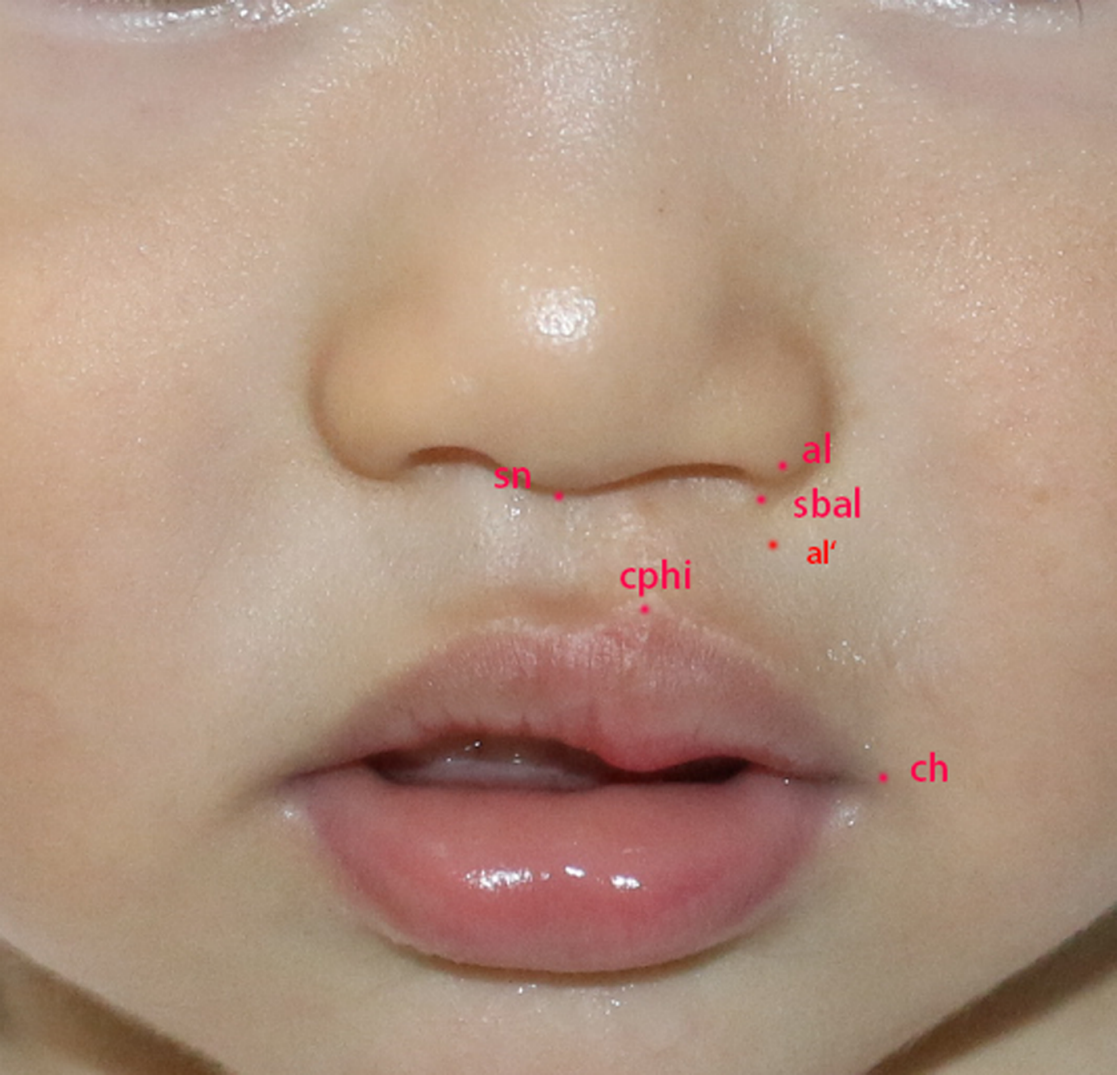
A patient with UCLP (right side) who underwent primary repair at 3 months of age showing the landmarks of anthropometric measurements. sn, the midpoint of the columellar base at which the lower border of the dorsal septum and the surface of the cutaneous upper lip meet; al, the most lateral point on each alar contour; al’, the dimension sn–al measured at sn–al’ along the perpendicular to the horizontal line through sbal; sbal, the medial point at the inferior limit of each alar base that is tangential to the cutaneous upper lip; cphi, the point on each elevated margin of the philtrum immediately above the vermilion line; ch, the point located at each labial commissure; sn–al, heminasal width; sn–cphi and sbal–cphi, labial height; cphi–ch, labial width; sn–sbal, nasal sill width . UCLP, unilateral cleft lip and palate.

Some facial anatomical landmarks were used to quantitatively assess nasolabial growth patterns, such as crista philtri (cphi), cheilon (ch), subnasale (sn), alare (al), and subalare (sbal) (15). Labial width (cphi–ch), heminasal width (sn–al), medial-oblique labial height (sn–cphi), lateral-oblique labial height (sbal–cphi), and nasal sill width (sn–sbal) are the most globally and widely used evaluation indices, and these were used in this study (16) (Figure 1). In addition, nasal tip angle, columellar angle, columellar–labial angle, columellar height, and dome height were used as evaluation parameters (17).

## 3. Lip

### 3.1. Labial width

Primary repair leads to significant improvements in the morphology of the upper lip and nose (18). In order to study the labial growth changes in UCLP patients, Ayoub et al. analyzed the changes in crista philtri in 21 patients with unilateral CL who underwent primary repair at the age of 3 months. At 3 years postoperatively, the crista philtri on both the cleft and the non-cleft sides was displaced laterally and posteriorly, and philtrum width (cphi–cphi’) and labial width (cphi–ch) increased significantly (19).

Mulliken and Labrie further measured and analyzed the labial width of 99 UCLP patients repaired by modified rotation advancement. The average labial width of the cleft side was about 8.36% shorter than that of the non-cleft side, but at a follow-up at 6 years of age, the gap was reduced to only 2.80%, which implied an asymmetrical growth pattern (16). Knight et al. re-evaluated a number of patients and extended the observation period, providing more details about the rate of change (20). They divided the follow-up periods into two phases: the first included all 99 patients from the operational age to an average age of 6.6 and the second involved a subset of 50 patients at an average age ranging from 6.6 to 11.5. They disclosed that immediately upon operation, the average labial width of the cleft side became 9.15% shorter than that of the non-cleft side, but the gap was narrowed to 4.39% and 2.75%, respectively, during the subsequent two follow-ups (20). Obviously, the labial width of the cleft side and the non-cleft side increased disproportionately, with a greater increase in the width of the cleft side during the growth phase. As time passed by, the gap in the labial width between the two sides gradually narrowed, and the labial width on the cleft side was closer to that on the non-cleft side.

The discordance in labial width will affect the sagittal and vertical positions of the philtrum, resulting in philtrum inclination to the cleft side after primary repair (16, 19). In patients with UCLP, the labial width on the non-cleft side increased at a slower pace than that on the cleft side, and the crista philtri on the cleft side will drift medially during the subsequent growth phase, gradually approaching the position that is almost symmetrical with that on the non-cleft side. Then, the philtrum on the cleft side will also rotate from the lateral inclination to the vertical direction (16, 20) (Table 1).

**Table 1:** Results of the main reviewed studies.

Reference	Country	Number of patients	Measurement methods	Patients age (years)	Follow-up	Associated procedures	Cleft type	Surgical techniques	Measurements
Ayoub et al. (19, 21)	Britain	21	3D images	3	Up to 3 years	McComb primary rhinoplasty	Unilateral (complete and incomplete)	Modified Millard cheiloplasty	Labial width and height; nasal base width; nasal height and projection; columellar length
Mulliken and Labrie (16)	United States	99	Direct anthropometry	6	Up to 6 years	Primary nasal correction	Unilateral (complete and incomplete)	Rotation advancement	Heminasal width; Labial height; labial width
Knight et al. (20)	United States	50	Direct anthropometry	11.5	Up to 11.5 years	Primary nasal repair	Unilateral (complete and incomplete)	Rotation advancement	Heminasal width; Labial height; labial width
Tse et al. (22)	United States	102	3D images	5	Up to 5 years	Foundation-based rhinoplasty	Unilateral (complete, incomplete and microform)	Cleft lip repair	Subalare; subnasale; alar base; nostril
Bell et al. (23)	Britain	95	3D facial models	10	Up to 10 years	McComb nose repair	UCLP (51); UCL (44)	Millard lip repair	Mean square distances between landmarks
Zreaqat et al. (24)	Malaysia	30	3D images	8–10	Up to 10 years	Not used	Unilateral (complete and incomplete)	Rotation advancement	Alar base root width; lip length; nose base/mouth width ratio
Chang et al. (25)	Taiwan	76	Photogrammetry	5	Up to 5 years	Primary rhinoplasty; nasoalveolar molding	Unilateral (complete and incomplete)	Modified rotation advancement (Mohler incision)	Nostril width and height; nasal sill height; nostril height-to-width ratio
Miyamoto and Nakajima (26)	Japan	31	3D computerized tomography	5–8	Up to early childhood	Primary rhinoplasty	Unilateral (complete and incomplete)	Rotation advancement	Nasal width; columellar length; nasal tip protrusion; vertical nasal tip projection
Moreira et al. (3)	Canada	70	Lateral cephalometric measurements	7–18	Up to 18 years	Not used	UCLP (complete)	Rotation advancement	Lip (nose) length and protrusion

UCLP, unilateral cleft lip and palate; UCL, unilateral cleft lip.

### 3.2. Labial height

Comparing the distance from the labialis superioris (the most prominent upper midline point of the vermilion border of the upper lip) to the subnasale with the labial height, Ayoub et al. found that there was no difference in the labial height between the operated CL patients and the non-cleft children at 3 years of life (19). This finding necessitated that labial height should be described in terms of two parameters, medial-oblique height (sn–cphi) and lateral-oblique height (sbal–cphi) (20).

Mulliken et al. disclosed that immediately after repair, the medial-oblique height on the cleft side became slightly longer and the lateral-oblique height became shorter, but 6 years later, the medial-oblique height eventually matched that on the non-cleft side, while the lateral-oblique height remained shorter. Thus, it could be speculated that the growth rate of the medial-oblique height on the cleft side was slower than that on the non-cleft side, whereas the growth rate of the lateral-oblique height was consistent on both sides (16). As evidenced by Knight et al., in the first phase (6.6 years), the medial-oblique height on the cleft side increased at a slower rate (5.2%) than that on the non-cleft side. In the second phase (11.5 years), the cleft and noncleft sides saw an almost equivalent growth at a rate of 3.53% and 3.02%, respectively. Specifically, the medial-oblique height on the cleft side was 7.14% longer than on the non-cleft side immediately upon operation, 2.08% longer at 6.6 years, and 2.61% longer at 11.5 years. The medial-oblique height on the cleft side increased at a slower pace initially but proportionately with the non-cleft side later on, finally increasing faster. As for the lateral-oblique height (sbal–cphi), it was on average 3.66% shorter on the cleft side than that on the non-cleft side immediately upon operation and remained significantly shorter on the cleft side by 3.12% at the age of 6.6 and by 2.47% at the age of 11.5 (20). There was no significant difference in the growth rate of the lateral-oblique height between the cleft side and the non-cleft side. In other words, the height of both sides increased equally all through the two follow-up periods.

Based on the above, and together with the rapid growth of the transverse labial width on the cleft side, it is meaningful to mark the lateral crista philtri on the cleft side closer to the commissure to ensure the restoration of the labial height on the non-cleft side (16) (Table 1). Generally, current methods of primary repair are able to restore labial height, labial width, and labial symmetry effectively.

## 4. Nose

### 4.1. Alar base

According to the anthropometric principle of Farkas, the malposition of the alare base has always been represented by the subalare (15, 27, 28). Traditional perspectives revealed that for a unilateral cleft lip, lateral and inferior deviation of the cleft alar base led to deformities. However, Tse et al. disputed this and certified upon a 3D image analysis that subalares on both sides were displaced, and compared with the cleft side, the non-cleft side alar base located more laterally to the facial midline. Surgical corrections involved an anterior rather than a horizontal movement of the subalare on the cleft side and an unexpected medial movement of the subalare on the non-cleft side considering that no dissection or suture was performed in that site (22) (Table 1). The position of the subalare on the cleft side in the sagittal direction was almost symmetrical to the non-cleft side immediately upon operation (16, 29, 30).

Three years after operation, the alar bases on the cleft side and the non-cleft side remained basically symmetrical, both drifting laterally and posteriorly but maintaining the vertical position (21). Even so, the nostril width (sbal–sbal’) on both sides was significantly lower than that preoperatively (31) (Table 1). In the following age of 8–10 years, the width between the alare (al–al’), the width between the subalare (sbal–sbal’), and the ratio of the width of the subalare to the labial width (Sbal–Sbal’/Cphi–Ch) were significantly higher than those in the non-cleft population (20, 24). At the age of 10, the subalare of the cleft side in patients with a cleft palate drifted significantly more laterally than in the cleft lip–only group and normal control group (23, 32). However, the symmetrical results of repair, including retrusion of the alar base, were consistent over time and were unrelated to the fact whether alveolar cleft bone grafting was performed or not (22).

The subalare on the cleft side will continuously drift laterally from the postoperative period to adulthood (16, 23, 33) (Table 1). Therefore, it should be overcorrected medially and fixed on the nasal muscle or periosteum during primary repair (13, 16).

### 4.2. Nostril

Nostril height and nostril width were the most common parameters used to evaluate the changes in nostril morphology. The asymmetry of the nose improved immediately postoperatively, and there was a reduction of the total nostril width postoperatively compared with the preoperative period (31). The nostril height ratio significantly increased 4 years after McComb primary rhinoplasty (0.89 vs. 0.58), while there was no significant increase in nostril width (34). Chang et al. on one occasion measured and analyzed the photographs of 76 patients with CLP who were approximately 5 years old. He found that patients who underwent primary rhinoplasty (overcorrection) during CL primary repair showed the best postoperative results: the nostril height ratio 5 years postoperatively was significantly higher than that in those who did not undergo primary rhinoplasty (0.95 vs. 0.77), and the nostril width ratio was lower (1.21 vs. 1.36) although without any statistical significance (25) (Table 1). At 18–25 years of age, clinical differences still existed in the nasolabial region, which mainly manifested as a wider, larger, or flatter nostril (35).

The postoperative outcomes of the nostril depended not only on the preoperative severity of clefts but also on primary rhinoplasty (32). Evidence implied that rhinoplasty in infancy would not impair nasal growth and development (17). Accordingly, several surgeons advocated rhinoplasty simultaneously with primary lip repair (12, 13, 16, 30) and suggested 20% overcorrection to optimize the nostril height (7, 16, 25).

### 4.3. Nasal tip, columella, and nasolabial angle

Cerrati and Dayan observed a coordinated change between the nasal tip and the upper lip projection. As the nasal tip protruded, the upper lip projection increased (36), which would definitely cause changes to the nasolabial angle. The morphology of the nasal tip and columella is known to be braced mainly by LLC, so changes in LLC would possibly affect the morphology of the columella (7, 29). Therefore, we will discuss these three parameters together in this section.

At the postoperative 10 months, there was a partial improvement in the nasal tip and it was restored to a near normal position, but it was still lower and dislocated in the vertical direction (37). Then, at the postoperative 4 years, the average length of the columella on the cleft side was longer than that immediately upon operation, and the nasolabial angle and columellar angle increased, with the nasolabial angle showing the most significant increase (110.03 ± 3.31 vs. 94.62 ± 2.73) (34). At 5–8 years postoperatively, all other nasal measurements were satisfactory except for the vertical tip position (26). From 7 to 17.9 years of age, the columellar length in CLP patients averagely remained short of 2 mm, exhibiting a reduction of the nasolabial angle, especially between 11.1 and 17.9 years (105.34 ± 2.80 vs. 100.36 ± 2.97) (3) (Table 1).

These nasal changes might be attributed to the re-establishment of the muscle balance on both sides. After that, the nasolabial angle and columella could be corrected by growth and development (10, 28). Since there was a lack of muscle in the area near the nasal tip, this region was susceptible to scar contracture, which resulted in a downward rotation of the columella and the nasal tip. In addition, nasal tip and columella deformities were caused by a displacement of the anterior nasal spine and caudal septum (13), and therefore, an overcorrection of the nasal tip to an extremely forward sagittal position would be necessary during primary repair to compensate for its depression caused by an insufficient bone base (12, 13).

### 4.4. Overall symmetry of the nose

The soft tissue morphology of the nose and lip in patients with UCLP is more asymmetrical than that in patients with BCLP (4, 38). At 3–4 months postoperatively, the overall symmetry of the nose significantly improves (18, 39). However, the nostrils in patients with complete UCLP (all repaired at 6 months of age) are still largely asymmetrical at 3 and 6 months postoperatively, and the nasal tip inclines toward the non-cleft side at 9 months of age. The amount of edema decreases by two-thirds at 1 month, 95% at 6 months, and 97.5% at 1 year after rhinoplasty (40). Therefore, the appropriate time to evaluate the nostril morphology should be at least 6 months postoperatively. At 12 months of age, the symmetry of the nose ameliorates, with the remnant asymmetry observed on the nostril rim (41). In patients aged 6–12 years after primary repair, the nasal soft tissue exhibits a better symmetry than the hard tissue, and this could be attributed to the compensatory growth of the nasal soft tissue, especially in the vertical and sagittal dimensions (28). It is also believed that at this age, despite the nasal symmetry being close to “normal,” the asymmetry persists in most patients (23, 42). In adulthood, however, assessed by 3D images in patients of the unilateral cleft lip–only group, no statistically significant asymmetry between the cleft side and the non-cleft side of the nose is identified (33, 43).

## 5. Summary

Surgical outcomes of cleft lip primary repair continue to improve with age, especially in the lip. However, the revision of nasal deformities remains a challenge, and this may be the last and most complicated aspect of cleft care, the reasons for which are traced to the multiple problems discussed in this review. Fortunately, primary rhinoplasty confirmed no significant impairment of the growth and development of the nose. Therefore, compared with secondary rhinoplasty, primary lip repair, combined with primary rhinoplasty, has more advantages in terms of establishing a new dynamic muscular balance around the nasolabial region, which may result in a more symmetrical nose during its growth and development, requiring less intervention at the time of definitive secondary rhinoplasty.
